# Distinct shape-shifting regimes of bowl-shaped cell sheets – embryonic inversion in the multicellular green alga *Pleodorina*

**DOI:** 10.1186/s12861-016-0134-9

**Published:** 2016-10-13

**Authors:** Stephanie Höhn, Armin Hallmann

**Affiliations:** 1 Department of Cellular and Developmental Biology of Plants, University of Bielefeld, Universitätsstr. 25, 33615 Bielefeld, Germany; 2 Present address: DAMTP, Biological Physics, University of Cambridge, Centre for Mathematical Sciences, Wilberforce Road, Cambridge, CB3 0WA UK

**Keywords:** Cell-cell connections, Cell sheet bending, Cell sheet folding, Cytoplasmic bridges, Evolution, Green algae, Inversion, Morphogenesis, Multicellularity, Volvocales

## Abstract

**Background:**

The multicellular volvocine alga *Pleodorina* is intermediate in organismal complexity between its unicellular relative, *Chlamydomonas*, and its multicellular relative, *Volvox*, which shows complete division of labor between different cell types. The volvocine green microalgae form a group of genera closely related to the genus *Volvox* within the order Volvocales (Chlorophyta). Embryos of multicellular volvocine algae consist of a cellular monolayer that, depending on the species, is either bowl-shaped or comprises a sphere. During embryogenesis, multicellular volvocine embryos turn their cellular monolayer right-side out to expose their flagella. This process is called ‘inversion’ and serves as simple model for epithelial folding in metazoa. While the development of spherical *Volvox* embryos has been the subject of detailed studies, the inversion process of bowl-shaped embryos is less well understood. Therefore, it has been unclear how the inversion of a sphere might have evolved from less complicated processes.

**Results:**

In this study we characterized the inversion of initially bowl-shaped embryos of the 64- to 128-celled volvocine species *Pleodorina californica*. We focused on the movement patterns of the cell sheet, cell shape changes and changes in the localization of cytoplasmic bridges (CBs) connecting the cells. The development of living embryos was recorded using time-lapse light microscopy. Moreover, fixed and sectioned embryos throughout inversion and at successive stages of development were analyzed by light and transmission electron microscopy. We generated three-dimensional models of the identified cell shapes including the localization of CBs.

**Conclusions:**

In contrast to descriptions concerning volvocine embryos with lower cell numbers, the embryonic cells of *P. californica* undergo non-simultaneous and non-uniform cell shape changes. In *P. californica*, cell wedging in combination with a relocation of the CBs to the basal cell tips explains the curling of the cell sheet during inversion. In volvocine genera with lower organismal complexity, the cell shape changes and relocation of CBs are less pronounced in comparison to *P. californica*, while they are more pronounced in all members of the genus *Volvox*. This finding supports an increasing significance of the temporal and spatial regulation of cell shape changes and CB relocations with both increasing cell number and organismal complexity during evolution of differentiated multicellularity.

**Electronic supplementary material:**

The online version of this article (doi:10.1186/s12861-016-0134-9) contains supplementary material, which is available to authorized users.

## Background

**Fig. 1:**
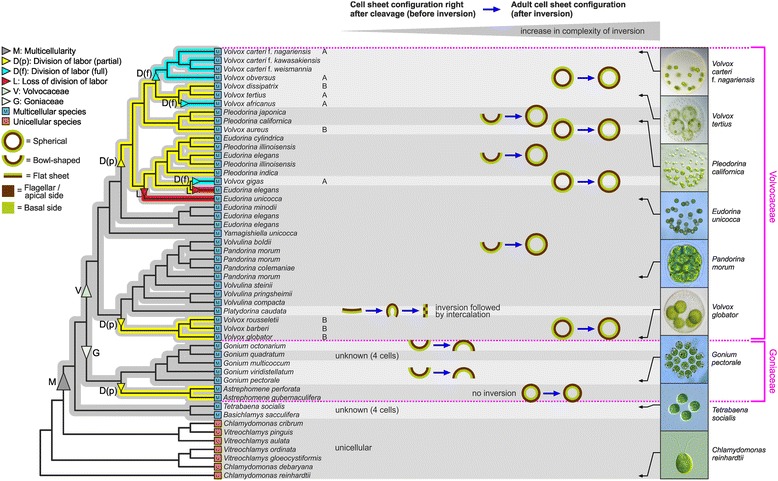
Schematic representations of cell sheet configurations of volvocine algae before and after embryonic inversion mapped on a phylogenetic tree. Blue arrows lead from the cell sheet configurations of embryos right after cleavage (before inversion) to the cell sheet configurations of adults (after inversion). The flagellar/apical side of the cell sheet is shown in brown color and the basal side of the cell sheet is shown in green color. Cell sheets of volvocine algae either are spherical, bowl-shaped or flat. Inversion processes with lower complexity are shown more to the left side and inversion processes with increased complexity are shown more to the right side. The background shading pools species with the same cell sheet configuration before and after embryonic inversion. Light micrographs on the right side of the figure show wild-type phenotypes of some representative volvocine species at adult stages. The evolutionary tree is based on the nucleotide sequences of five chloroplast genes. The phylogenetic analysis indicates that multicellularity evolved only once in this group. In contrast, a partial germ-soma division of labor evolved independently in three different lineages and was lost twice [3, 6, 8, 84, 107]. A full germ-soma division also evolved three times. There are two fundamentally different sequences through which embryos of the genus *Volvox* turn right-side out: type A and type B inversion [38, 108]. Letters A or B behind names of *Volvox* species indicate which inversion sequences embryos of these *Volvox* species undergo (type A or B). The meanings of symbols are given at the left edge of the figure. This tree was adapted from Herron and Michod [6] and others [3, 8, 35, 55] and some additional information was added [38, 56, 57]

Phylogenomics and relaxed molecular clock analyses showed that in plants, fungi and animals multicellularity evolved more than half a billion years ago [1] and some fossils even indicate that simple multicellular organisms existed as early as two billion years ago [2]. The molecular origins of multicellular life are therefore obscured by hundreds of millions of years of evolution. In contrast, multicellular volvocine green algae diverged relatively recently from their unicellular relatives: the last common ancestor of the unicellular alga *Chlamydomonas reinhardtii* and the multicellular alga *Volvox carteri* lived just about 200 million years ago [3]. The volvocine algae form a group of genera closely related to the multicellular genus *Volvox* within the order Volvocales (Chlorophyta) (Fig. 1, Additional file 1). However, *Volvox* is the only volvocine genus in which a complete division of labor between (many) biflagellate somatic cells and (a few) non-motile reproductive cells exists. In other multicellular volvocine genera, relatively few reproductive cells are derived from biflagellate cells that originally look and function like somatic cells before they enlarge and divide to form new progeny. One example of this is *Pleodorina*, which is intermediate in organismal complexity between *Volvox* and its unicellular volvocine relatives (e.g., *Chlamydomonas* and *Vitreochlamys*) (Fig. 1, Additional file 1). The differences in organismal complexity between volvocine algae include, for instance, the cell number, the grade of germ-soma differentiation, the complexity of cell sheet deformations and the grade of extracellular matrix expansion (Additional file 1) [4–8]. This situation makes the volvocine algae an excellent model to study the transition from unicellularity to multicellularity with division of labor between different cell types. The mode of cell division of most asexually reproducing volvocine algae is known as palintomy and multiple fission [9, 10]. For this mode of cell division, asexual reproductive cells grow 2^n^-fold in size and then divide rapidly n times by multiple fission to produce 2^n^ offspring cells. The n has a value from 2 to 15 depending on the volvocine species and, to some extent, on the environmental conditions [4, 11]. In *C. reinhardtii*, the n is usually 2 or 3, in *Pleodorina californica* it is 6 or 7 and in *V. carteri* it is usually 11 or 12. In multicellular volvocine species, offspring cells stay linked to each other by cytoplasmic bridges throughout the rest of embryogenesis due to an incomplete cytokinesis [12–18].

*P. californica* consists of 64 to 128 biflagellate cells at the surface of a transparent sphere of glycoprotein-rich extracellular matrix (ECM) with a diameter of 100–300 μm (Fig. 1, Additional file 1) [19–21]. In *Pleodorina*, all cells of the anterior hemisphere remain small and function as non-dividing somatic cells, while posterior cells eventually grow and divide. In the smaller multicellular relatives of *Pleodorina*, which consist of only 8 to 32 cells, all cells have reproductive capabilities. The adults of those multicellular volvocine genera with 8 to 32 cells are either organized as almost flat or bowl shaped cell sheets (*Platydorina* [22, 23]*, Gonium* [18, 24–27]) or as small spheroids (*Pandorina* [28]*, Volvulina* [29, 30]*, Eudorina* [16, 31]*, Yamagishiella* [21]) (Fig. 1, Additional file 1). The larger multicellular relatives of *Pleodorina* are species of the genus *Volvox* (Fig. 1, Additional file 1). These spheroidal algae feature the highest cell numbers, ranging from several thousand to 50,000 cells. They possess mostly somatic cells arranged in a monolayer at the surface and a much smaller number of germ cells. *V. carteri, V. obversus, V. tertius*, *V. gigas*, and *V. africanus* [4, 32–35] exhibit full germ-soma differentiation [35], i.e., they show a complete division of labor between the numerous somatic cells and some asexual reproductive cells.

David Kirk suggested twelve morphological and developmental changes that are believed to be required for the transition from a *Chlamydomonas*-like unicellular ancestor to the multicellular species *Volvox carteri* with its two cell types [36]. The first changes were the occurrence of incomplete cytokinesis, the transformation of cell walls of unicells into an extracellular matrix embedding multiple cells, all of which maintaining reproductive capabilities, and the genetic control of the maximum number of cells per organism. As a result of incomplete cytokinesis, the embryonic cells are linked to one another by cytoplasmic bridges (CBs) but so far it is unknown whether the CBs are merely structural components or whether they also function in cell-cell signaling.

Another essential step towards multicellularity was the evolution of a mechanism for cell sheet folding, which is required in multicellular volvocine embryos to turn themselves right-side out at the end of embryogenesis and to expose their flagella. This process, in which the orientation of the cell sheet is reversed and the embryos achieve their adult configuration, is called ‘inversion.’ After the completion of the cell division phase and before inversion, the embryos of *Gonium* [18, 26]*, Pandorina* [37, 38]*, Eudorina* [16, 38] and *Pleodorina* [20] consist of a bowl-shaped cell sheet, whereas the embryonic cells of *Volvox* [38, 39] form a spherical cell sheet. With exception of the genus *Astrephomene* [40–42], all multicellular volvocine embryos face the same “problem”: the flagellar ends of all the cells point toward the interior of the bowl-shaped or spherical cell sheet rather than to the exterior, where they need to be later to function during locomotion. The correction of this awkward situation by inversion has been investigated in some multicellular volvocine genera with different degrees of detail [4, 16–18, 20, 23, 37–39, 43–54].

**Fig. 2:**
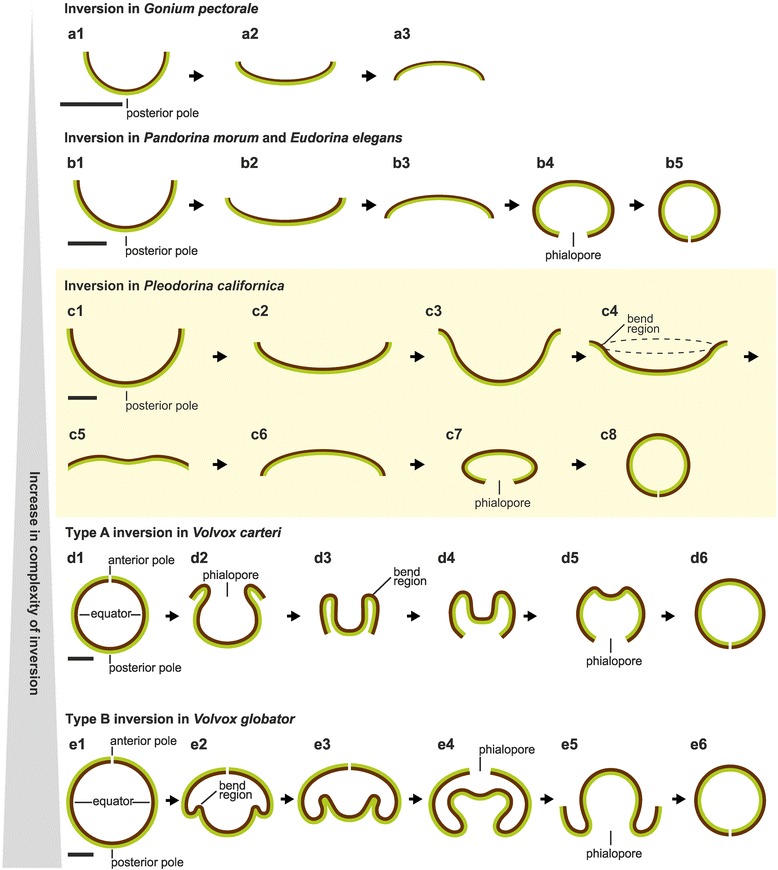
Stylized sequences of inversion processes in different multicellular volvocine species. Schematic representation of midsagittal cross sections of embryos of (**a**) *Gonium pectorale* [18, 38], (**b**) *Pandorina morum* [37, 38] and *Eudorina elegans* [16, 38], (**c**) *Pleodorina californica* [20], (**d**) *Volvox carteri* [38, 39, 51] and (**e**) *Volvox globator* [38, 53]. It should be noted that the overall deformation of the cell sheet of *P. californica* in (**c1-c8**) has been described previously by Kikuchi [20]. However, Kikuchi mentioned pre-inversion movements in stage (**c2**), which have never been observed in our studies (see main text). **a**-**e** The side of the cell layer that is outside in the adult configuration and from which the flagella will emerge is presented in brown, and the side of the cell layer that is inside in the adult configuration and where the reproductive cells are located, is presented in green. Light micrographs of several volvocine species at adult stages are shown in Fig. 1 and in Additional file 1. Scale bars: (**a-c**) 10 μm; (**d, e**) 20 μm

The 8- to16-celled embryos of *Gonium*, which are bowl-shaped before inversion, change their shape during inversion from concave to slightly convex (with respect to the apical or flagellar cell ends) (Fig. 2a). Kirk termed this form of inversion incomplete because the convex cell sheet does not bend further to obtain an ellipsoid or spherical shape [36]. Bowl-shaped embryos with more cells, like those in *Pandorina, Eudorina* and *Pleodorina*, likewise change their shape from concave to slightly convex but then they progressively become highly convex and, finally, ellipsoid or spherical (Fig. 2b, c). Therefore, species like *Pandorina*, *Eudorina* and *Pleodorina* show a complete inversion [16, 20, 36, 38]. Embryos in the genus *Volvox* also undergo a complete inversion, but a difference is that embryos are even spherical before inversion. These initially spherical embryos turn completely inside out and finally regain their spherical shape [38]. However, the tactic for turning the spherical cellular monolayer right-side out varies between different *Volvox* species and there are two fundamentally different sequences of inversion processes: type A and type B [4, 17, 38, 39, 45–51] (Fig. 1). Type A inversion proceeds as follows: (i) An opening of the cell sheet with four lips forms at the anterior pole of the embryo, the ‘phialopore’; the four lips curl backwards. (ii) The cell sheet moves over the subjacent cell layer, i.e., the anterior hemisphere inverts. (iii) The posterior hemisphere contracts. (iv) The posterior hemisphere inverts and, finally, (v) the phialopore closes (Fig. 2d). The movement pattern in type B inversion appears in this way: (i) The posterior hemisphere contracts while a circular invagination appears at the equator of the embryo. (ii) The posterior hemisphere moves into the anterior hemisphere while gradually inverting. (iii) The phialopore opens and widens. (iv) The anterior hemisphere moves over the subjacent posterior and inverts. (v) Finally, the phialopore closes (Fig. 2e). The genus *Volvox* is known to be polyphyletic and its typical body plan and development with initially spherical embryos evolved multiple times, i.e., it is a matter of convergent evolution. It has been discussed controversially, whether in *Volvox* the type A or type B sequence of inversion evolved first [6, 38, 53]. In order to provide an overview, we mapped schematic representations of cell sheet configurations of volvocine algae before and after embryonic inversion on the phylogenetic tree in Fig. 1 [6, 35, 55–57].

Inversion has been suggested as a model system to study the basic principles that underlie cell sheet bending or folding in multicellular organisms [38, 39, 53, 58]. In Metazoa, this active process is required for normal morphogenesis in many instances of multicellular embryogenesis, including gastrulation, neurulation and organogenesis (e.g., optic cup-formation) [58–60]. Cell shape changes often play a major role in generating the forces that deform epithelia. Apical constriction leads to cell wedging that can eventually drive invagination of a cell sheet. Examples include the blastopore formation and neurulation in amphibians, the development of the primitive groove in birds and the formation of the ventral furrow in *Drosophila* [61–69]. The underlying mechanism causing cell shape changes is an active, three-dimensional reconstruction of the cytoskeleton. The forces produced by the reconstructing cytoskeleton are then propagated to neighboring cells since the cells of the cell sheet are connected to each other by adhesion molecules. The interaction of self-deforming, connected cells results in a global three-dimensional shape-shifting of a cell sheet or tissue. However, cell sheet folding in animal model organisms frequently involves other processes such as cell division, migration and intercalation, which obscure the specific role of cell shape changes [62–67, 70–80].

In contrast, the aforementioned inversion of embryos in multicellular volvocine algae provides a simple, straightforward model system for studying the role of cell shape changes in the dynamic morphology of cell sheets without influences from other processes. This is because in multicellular volvocine embryos the cell division phase is completed before the beginning of inversion and the cells do not change their position relative to their neighbors in the network. However, just as in Metazoa, self-deforming, connected cells transmit forces to neighboring cells by means of cell-cell-connections and the interaction of many cells allows for the coordinated bending of the cell sheet during inversion.

Previous studies of type A inversion in *V. carteri* revealed radially symmetrical waves of active cell shape changes that move across the inverting embryo. In addition, active movements of cells relative to the CBs connecting them have been observed. Both processes together are necessary for the successful progress and completion of inversion as shown by characterization of wild-type, mutant and chemically treated embryos [4, 17, 32, 33, 39, 43, 45–48, 50, 51, 81–84]. Similar cell shape changes in conjunction with a relocation of CBs have been reported for type B inversion [53, 54]. Inversion starts when cells around the phialopore of type A embryos or at the equator of type B embryos become wedge-shaped (i.e., flask or paddle-shaped, respectively) by developing narrow basal stalks. While developing stalks these cells are simultaneously moving relative to the network of CBs until they are connected at the tips of their stalks [51, 53]. This cell movement is mediated by a motor protein, the kinase InvA, which is associated with the cortical microtubule cytoskeleton. InvA is believed to be firmly connected to a structure within the CBs. Thus, when InvA moves along the microtubules, it pulls the cells towards the interior of the cell sheet [51].

By virtue of their geometries, it could be assumed that inversion of a bowl-shaped embryo (e.g., of *Pleodorina*, Fig. 2c) is less complex than the inversion of a spherical embryo of *Volvox* (Fig. 2d, e). However, there are no detailed descriptions of the inversion processes of genera with cell numbers of 64 to 128. In fact only a single previous paper describes the inversion process in *P. californica* [20]. Moreover, this paper by Kazuko Kikuchi is exclusively based on results from transmitted light microscopy. Kikuchi merely describes the shape of embryonic cells before and after inversion as “conical” without mentioning any cell shape changes during inversion [20]. Even though a homolog of InvA has been identified in *P. californica* [36], the question remained whether a relocation of CBs occurs during the curling of the embryonic cell sheet that could be crucial for inversion in this species.

In this study we analyze the inversion process of the bowl-shaped embryos of *P. californica.* We provide a detailed characterization of cell shape changes, cell sheet deformations and the relative position of cell-cell connections (cytoplasmic bridges, CBs). For this purpose, we use time-lapse transmitted light microscopy, light microscopy of semi-thin sections and transmitted electron microscopy of thin sections. The observed cell sheet deformations, shape changes and relocations of the CBs are integrated into a summary model for epithelial folding in *Pleodorina*. In the ensuing discussion, we address details of our model including the role of intra- and inter-cellular forces and biomechanical constraints. Furthermore, we discuss the inversion process of *Pleodorina* and its relatives from an evolutionary perspective. Our results will be valuable for future comparative studies reconstructing the phenotypic or developmental evolution of volvocine algae.

Moreover, the bending of cell sheets plays a major role in in early animal development including gastrulation, neurulation and organogenesis, but there it often arises from a complex and poorly understood interplay of cell shape changes, division, migration and intercalation. A better understanding of the comparatively simple cell sheet folding in *Pleodorina* and its relatives can also improve the understanding of cell sheet bending and folding in animal development.

## Results

**Fig. 3:**
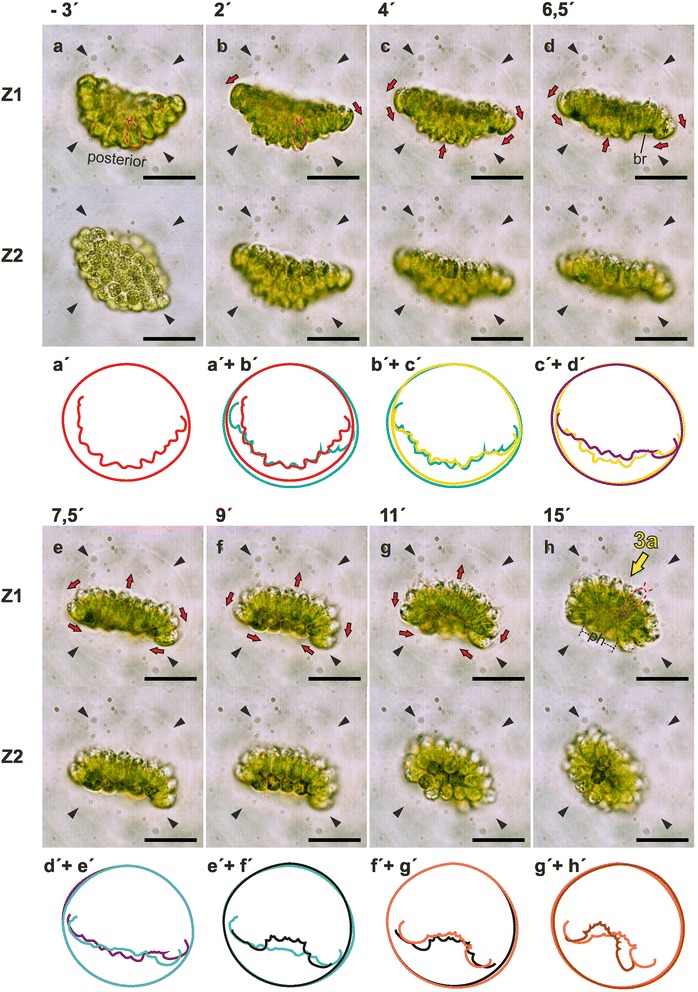
Sequence of in vivo light micrographs and traced contours of an inverting *P. californica* embryo. In vivo time-lapse sequence of an embryo within its embryonic vesicle (black arrowheads) after mechanical separation from its mother spheroid. **a-h** lateral view; upper panel (Z1): medial focus plane; middle panel (Z2): peripheral focus plane; lower panel: traced contours of the embryo and its embryonic vesicle. The contours of the respective time point and the preceding time point were superimposed (consequently, there is only a single contour at the first time point). **a-h** Points in time are given above each column. The start of inversion is at time zero. Red arrows: direction of cell sheet movements. **a** Before inversion begins the embryo is bowl-shaped and all cells are teardrop-shaped (pink dashed line, also see Fig. 5a). **b, c** Inversion begins with an outward curling of the peripheral cells; this movement deforms the embryonic vesicle. The apical ends of the peripheral cells become hemispherical, while their basal cell ends become thinner. The cells at the posterior pole of the embryo become spindle-shaped (dashed red line, see also Fig. 5c). **d, e** br: bend region; asterisk: bent peripheral cell. The posterior pole moves towards the opening of the cell sheet while the peripheral region continues to curl until the plakea is nearly flat. **f-h** ph: phialopore. The entire cell sheet proceeds to curl so that the previously concave plakea becomes convex. Simultaneously, all cells elongate and adopt an elongated teardrop shape (*pink dashed line* in **h**); a transparent chloroplast-free region appears at the apical part of each cell. Scale bars: 20 μm

**Fig. 4:**
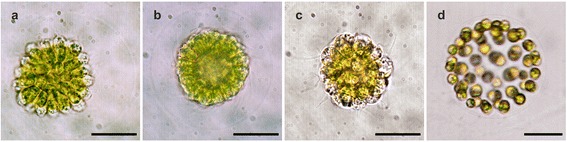
In vivo light micrographs of *P. californica* juveniles after completion of inversion. **a** Juvenile alga one minute after completion of inversion rotates within its embryonic vesicle; it is the same organism as the one shown in Fig. 3; anterior view. All cells have an elongated teardrop shape. Due to the different angle to the viewer, the elongated teardrop-shape cells in the center show almost hexagonal contours. **b** Juvenile about 30 min after completion of inversion; posterior view; the phialopore is still visible; the cells feature trapezoid contours (pink dashed line). **c** Juvenile about 40 min after completion of inversion. The cells are rounded, the phialopore is closed and the juvenile alga has adopted a spherical shape. **d** Juvenile shortly after release from its mother spheroid. The spherical cells already produced a significant amount of transparent ECM. Scale bars: 20 μm

**Fig. 5:**
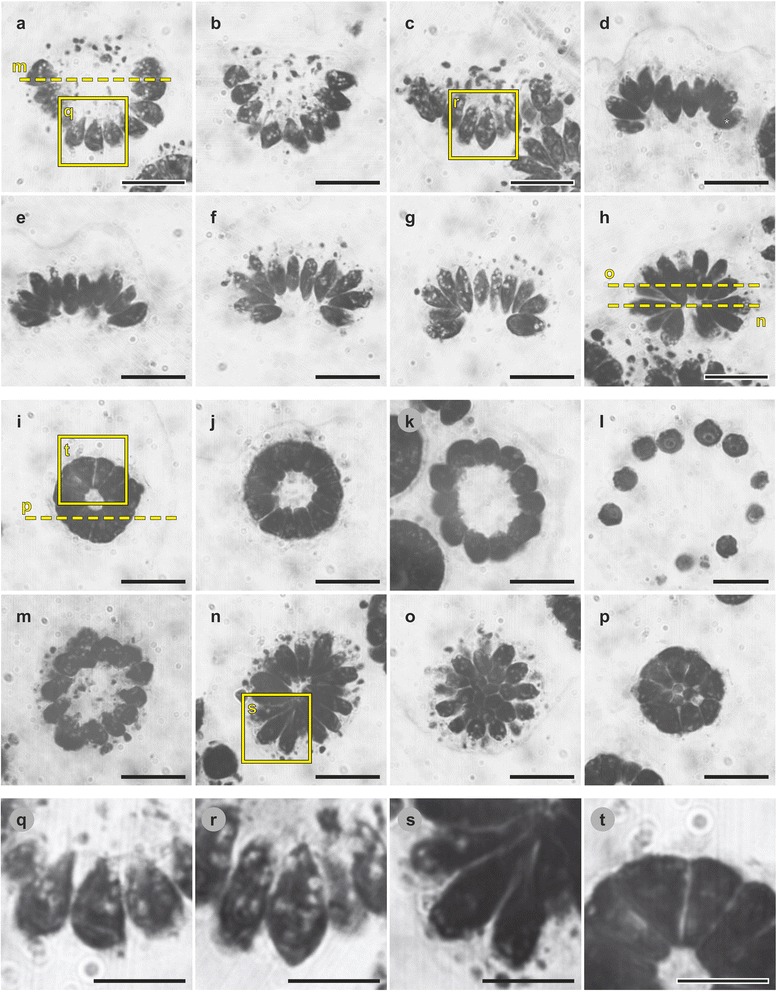
Sections of *P. californica* embryos during inversion and subsequent development. **a**-**t** In situ light micrographs of toluidine blue stained semi-thin sections of fixed embryos inside their mother spheroids. **a**-**h** Mid-sagittal sections of successive stages of inversion. The start of inversion is at time zero (t = 0 min). **a** t = −3 to −5 min before the beginning of inversion. **b** t = −0.5 to −1 min. **c** t = 3 to 4 min after the beginning of inversion. **d** t = 6 to 6.5 min; asterisk: bent peripheral cell. **e** t = 7 to 8 min. **f** t = 10 to 11 min. **g** t = 12 to 13 min. **h** t = 14 to 16 min; end of inversion. **i-l** Transverse sections of stages after completion of inversion. **i** t = 20 to 25 min after completion of inversion. **j** t = 25 to 30 min after completion of inversion. **k** t = 40 to 50 min after completion of inversion. **l** Juvenile shortly after release from its mother spheroid. **m-p** Sections at right angles (transverse) to the image planes shown in (**a**, **h**, **i**); the positions of the image planes for (**m-p**) are indicated in (**a**, **h**, **i**), respectively (dashed yellow lines). **m** t = −3 to -5 min. **n** t = 14 to 16 min. **o** t = 14 to 16 min. **p** t = 30 to 40 min. **q-t** Magnified images of the framed areas in (**a**, **c**, **I**, **n**), respectively. **q** Teardrop-shaped cells. **r** Spindle-shaped cells. **s** Elongated teardrop-shaped cells. **t** Truncated pyramid-shaped cells. Scale bars: (**a-p**) 20 μm; (**q-t**) 10 μm

**Fig. 6:**
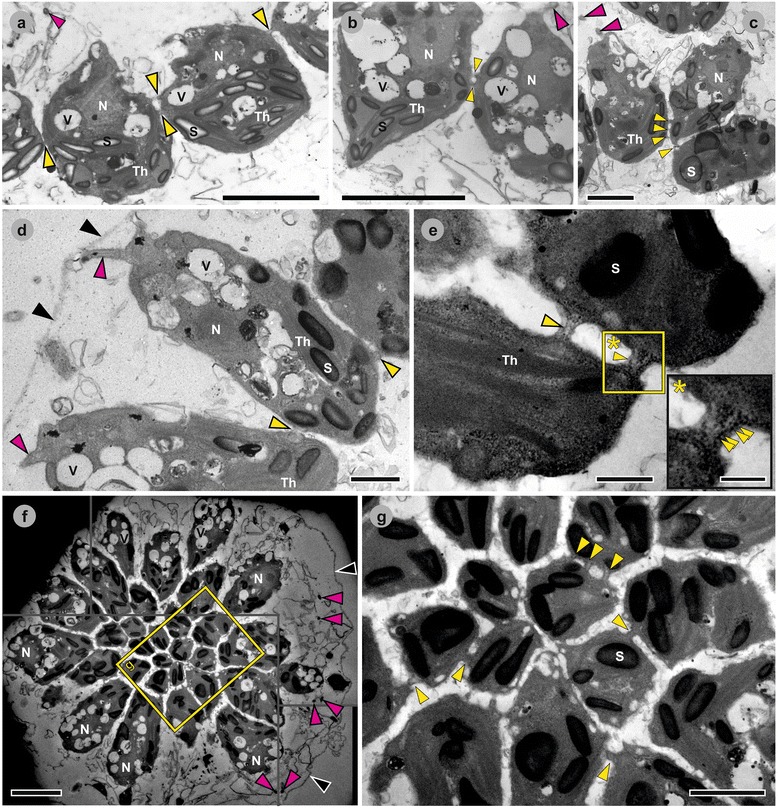
Transmission electron microscopy images of *P. californica* embryos during inversion. Heavy metal-stained sections of fixed *P. californica* embryos during inversion (inside their mother spheroid). **a** Midsagittal section; cells of an embryo at 3 to 5 min before inversion; apical cell ends face towards the inner surface of the plakea; CBs are located at the equator of the cells or within the apical half of the cells. **b** Midsagittal section; cells of an embryo at the beginning of inversion; note the pointed basal end of the left cell; CBs are located at the cell equator. **c** Sagittal section; peripheral cells of an outwards curled plakea at about 8 min after beginning of inversion; note the conical basal end of the cell in the middle (bend region); CBs are located at the basal cell ends. **d** Sagittal section; elongated cells of an embryo at about 15 min after the beginning of inversion; the flagella face outwards; CBs are located at the basal cell ends. **e** Sagittal section; basal cell ends of an embryo at about 15 min after the beginning of inversion; inset: rough endoplasmatic reticulum pervades the CBs. **f** Section of an embryo at about 15 min after the beginning of inversion; the cells are elongated and wedge-shaped; the flagella face outwards; center of the image: transversally sectioned basal cell ends of central cells of the plakea showing numerous CBs; image periphery: longitudinally sectioned peripheral cells of the plakea. **g** Magnified image of the framed area in (**f**). *Black arrowheads*: embryonic vesicle; *yellow arrowheads*: CBs; *magenta arrowheads*: flagella; ER: endoplasmatic reticulum, N: nucleus; S: starch grain; Th: thylakoids; V: vesicle-like structure. Scale bars: (**a**, **b**, inset in **e**) 500 nm; (**c**, **d**, **g**) 2 μm; (**e**) 1 μm; (**f**) 5 μm

**Fig. 7:**
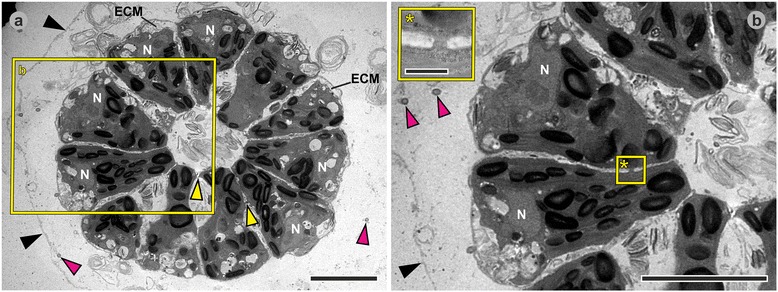
Transmission electron microscopy images of *P. californica* juveniles 20 to 25 min after completion of inversion. Heavy metal-stained sections of fixed *P. californica* juveniles 20 to 25 min after completion of inversion (still inside the mother spheroid). **a** Transverse section; cells with trapezoid contours; CBs are located at the basal halves of the cells; the beginning of ECM biosynthesis is already visible at the outer edge of some cells. **b** Higher magnification of boxed area in **a**; inset: even higher magnification of boxed area in the main image showing a single CB. *Black arrowheads*: embryonic vesicle; *yellow arrowheads*: CBs; *magenta arrowheads*: flagella; ECM: extracellular matrix; N: nucleus. Scale bars: (**a**, **b**) 5 μm; (inset in **b**) 2 μm

**Fig. 8:**
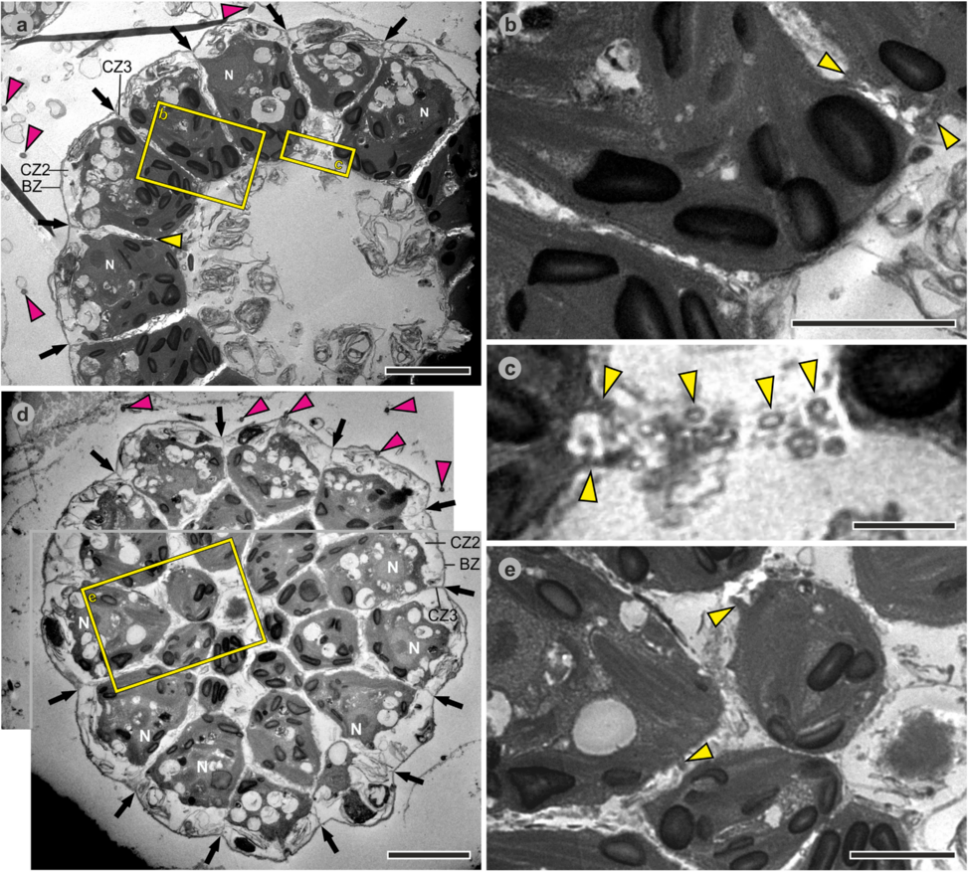
Transmission electron microscopy images of *P. californica* juveniles 25 to 35 min after completion of inversion. Heavy metal-stained sections of fixed *P. californica* juveniles 25 to 35 min after completion of inversion (still inside the mother spheroid). **a-e** Transverse sections. **a** Juvenile alga 25 to 30 min after completion of inversion; CBs are located at the basal ends of the cells; the cells are separated from each other by electron dense ECM material; the ECM zones boundary zone (BZ), cellular zones 2 and 3 [85, 86] are labeled. **b** Magnified image of the framed area in (**a**); CBs are located at the basal ends of the cells. **c** Magnified image of the framed area in (**a**); transverse section of CBs. **d** Juvenile alga 30 to 35 min after completion of inversion; center of the image: transversally sectioned basal cell ends showing numerous CBs; image periphery: longitudinally sectioned cells; the ECM zones boundary zone (BZ), cellular zones 2 and 3 (CZ2 and CZ3) are labeled. **E** Magnified image of the framed area in (**d**) showing CBs. *Yellow arrowheads*: CBs; *magenta arrowheads*: flagella; *black arrows*: electron dense ECM; N: nucleus. Scale bars: (**a**, **d**) 5 μm; (**b**, **c**, **e**) 2 μm

**Fig. 9:**
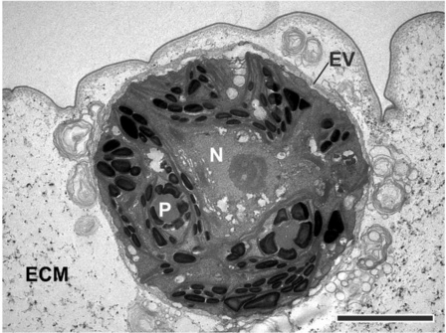
Transmission electron microscopy image of a single cell of a *P. californica* young adult shortly after release from its mother spheroid. Heavy metal-stained section of a *P. californica* young adult, which was fixed a short time after release from its mother spheroid; the image shows a picture detail with a single cell at high magnification; the spherical cell is enclosed in a newly synthesized embryonic vesicle (EV) and it is embedded in a considerable amount of extracellular matrix (ECM) material. N: nucleus; P: pyrenoid. Scale bar: 5 μm

**Fig. 10:**
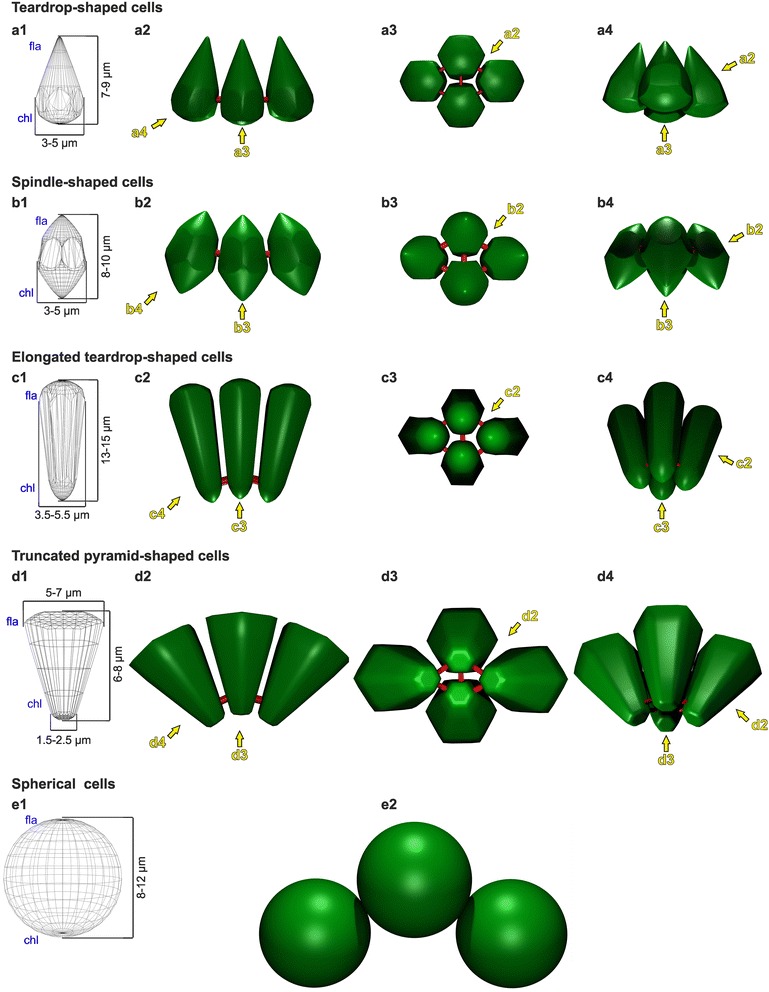
Surface-rendered three-dimensional representation of cell shapes and localization of CBs observed before, during and after embryonic inversion of *P. californica*. All cell shapes are radially symmetrical around their apical–basal axis. a1, b1, c1, d1, e1 Wireframe models; cell sizes are indicated; fla: flagellar/apical cell end; chl: chloroplast/basal cell end. a2, a3, a4, b2, b3, b4, c2, c3, c4, d2, d3, d4, e2 Textured models; groups of cells captured from different angles. Green: cell surfaces; red: CBs. Arrows indicate the angle of view in other images of the same cell shape. a2-e2 Frontal side view. a3-d3 View from below. a4-d4 Slanted side view. a1-a4 Teardrop-shaped cells: conical apical cell ends; hemispherical basal cell ends; transversal cross-sections at the basal cell ends have hexagonal outlines; CBs are located at the basal halves of the cells. b1-b4 Spindle-shaped cells: conical apical and basal cell ends; transversal cross-sections at the cell equators have hexagonal outlines; CBs are located near the cell equators. c1-c4 Elongated teardrop-shaped cells: cells are elongated compared to the teardrop and spindle-shaped cells and have thinned basal cell ends; transversal cross-sections along their entire length have hexagonal outlines; CBs are located close to the basal cell ends. d1-d4 Truncated pyramid-shaped cells: cells have expanded apical cell ends and thinned basal cell ends; both cell ends are flattened; longitudinal cross-sections have trapezoid outlines; transversal cross-sections along their entire length have hexagonal outlines; CBs are located near the basal cell ends. e1-e2 Spherical cells. The cells no longer have CBs and the distance between the cells increased due to ECM secretion

**Fig. 11:**
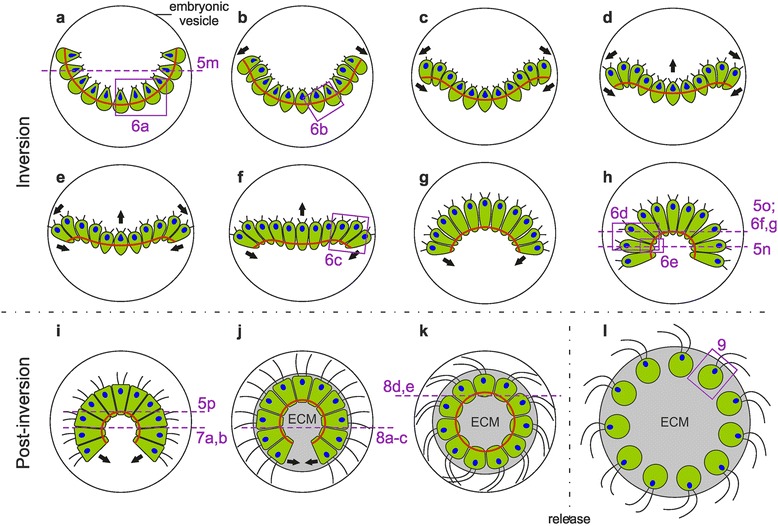
Model of the inversion process and subsequent development in *P. californica*. A schematic presentation of inversion as deduced from time-lapse transmitted light microscopy, light microscopy of semi-thin sections and transmitted electron microscopy of thin sections. Mid-sagittal cross-sections of an embryo of *P. californica* during inversion and subsequent development. Cell content is shown in green; red lines indicate the position of the CB network; nuclei are shown in blue. The directions of the cell layer movements are indicated by black arrows. Frames show the approximate localization of the details presented in Figs. 5, 6, 7, 8, and 9. **a** Pre-inversion stage. **b**, **c** Early inversion stage: the peripheral (anterior) region of the plakea bends outwards. **d-f** Mid-inversion stage: Bending of the peripheral region of the cell sheet continuous and, simultaneously, the centrally located posterior region of the plakea moves towards the opening of the bowl-shaped cell sheet. **g**, **h** Late inversion stage: the entire cell sheet proceeds to bend so that the previously concave plakea becomes more and more convex; at the end of inversion the cell sheet is two-thirds closed. **i-k** Post-inversion stage: the cells round up and the opening (phialopore) closes; ECM biosynthesis begins. **l** Young adult shortly after release from its mother spheroid; the cells are embedded in a considerable amount of ECM material


The cell sheet dynamics of the bowl-shaped embryos of *P. californica* were analyzed throughout inversion and during the subsequent development. A short movie in Additional file 2 gives an overview of the entire inversion process. The major movement patterns of the cellular monolayer, region and stage-specific cell shapes and the localization of CBs were characterized. For reasons of clarity, we first describe the movement patterns of the entire cell sheet throughout inversion in chronological order and then, in a second pass through the inversion process, we take a close look at the successive cell shape changes. To that end, we refer to the relevant panels of Figs. 3, 4 and 5, which show light micrographs of either intact, living organisms (Figs. 3 and 4) or semi-thin sections of chemically fixed organisms (Fig. 5) in successive stages of inversion and thereafter. Finally, we describe the localization of CBs (and smaller cellular changes) during inversion and thereafter while referring to the corresponding transmission electron microscopy images in Figs. 6, 7, 8 and 9. Three-dimensional models of the identified cell shapes are shown in Fig. 10 while Fig. 11 summarizes the results in a cellular model of the inversion process in *P. californica*. Furthermore, Fig. 11 contains cross references to the approximate in situ localization of the details shown in Figs. 5, 6, 7, 8, and 9.

Additional file 2: Short movie showing an inverting *P. californica* embryo. (AVI 1644 kb)


### Major movements of the cellular monolayer

#### Pre-inversion stage

In *P. californica* the mature, asexual reproductive cells, called “gonidia”, undergo 5 to 7 successive cell divisions to form an embryo. The cleavage phase is completed before inversion begins. A fully cleaved *P. californica* embryo consists of 64 or 128 cells arranged in a bowl-shaped cellular monolayer with a diameter of approximately 40 μm (Figs. 1 c1, 3a and 5a). Such a sheet of cells is called “plakea”. The apical ends (flagellar ends) of the cells point towards the interior of the bowl-shaped plakea, whereas the basal cell ends face the exterior (Fig. 5a and q). Each plakea is surrounded by an embryonic vesicle (Fig. 3), which was shown to be composed of glycoproteins in *Pleodorina’s* close relative *Volvox* [52].

At the stage after the completion of cell division and right before inversion Kazuko Kikuchi described a transition from a bowl-shaped plakea to an almost flat cell sheet that would then move back to its original bowl-shape (Fig. 2c) [20]. However, in our in vivo time-lapse recordings of numerous independent embryos we never saw such a reversible deformation of the plakea before inversion.

#### Inversion and subsequent development

In *P. californica* the inversion process starts when the peripheral region of the plakea (Fig. 3b-c; a´ + b´, b´ + c´) begins to curl outwards. This curling creates a circular region of maximum curvature that we term “bend region” (Fig. 2 c4). The outward curling of the plakea also somewhat deforms the embryonic vesicle (Fig. 3 a’ + b’). About 4.5 min after the beginning of inversion, the centrally located posterior region of the plakea begins to move towards the opening of the bowl-shaped cell sheet (Figs. 3c-d, c’ + d’ and 5c). The curvature of the peripheral region of the cell sheet increases while the posterior pole continues to move towards the opening until the plakea resembles a nearly flat cell sheet (Figs. 3e; d’ + e’ and 5d-e). Subsequently, the entire cell sheet proceeds to curl so that the previously concave plakea becomes more and more convex (Figs. 3f-h, e’ + f’, f’ + g’, g’ + h’ and 5f-h). It should be noted that the terms concave and convex refer to the localization of the apical ends (flagellar ends) of the cells. The cell sheet reaches its maximally convex curvature about 15 min after the beginning of the inversion process. At this stage the cellular monolayer has an elliptic outline when viewed from the side (lateral view, Fig. 3h), circular or elliptic (Figs. 4a and 5n) outlines when viewed in anterior-posterior direction and it has a circular opening, the phialopore, with a diameter of approximately 15 μm at the posterior pole (Figs. 3h and 5h). No further cell sheet movements are observable within the following 15 min. However, these juvenile algae frequently rotate within their embryonic vesicles due to their now exposed and fully functional flagella. For illustration, the Figs. 3h and 4a show one and the same rotating embryo in lateral and anterior view, respectively. After completion of inversion the posterior opening remains visible for 30 to 60 min (Fig. 4b). The opening closes during the subsequent development and the juvenile algae adopt a spherical shape (Fig. 4c). The juvenile spheroids then grow in volume due to a continuous secretion of glycoprotein components by the cells resulting in an expansion of the transparent ECM. Shortly after release from their mother spheroid, the young individuals are 50 to 60 μm in diameter (Fig. 4d).

### Region and stage specific changes in cell shape

#### Pre-inversion stage (Fig. 3a)

Before inversion starts, all cells within a plakea of *P. californica* exhibit a teardrop-shape with conical apical cell ends and hemispherical basal ends (Figs. 3a, 5a, m, q, 6a and 10a). These teardrop-shaped cells have a length of 7 to 9 μm along their apical-basal axis and a maximum width of 3 to 5 μm. The view onto the basal cell ends reveals approximately hexagonal or pentagonal outlines of the cells, which are closely packed (lower panel of Fig. 3a). Short flagella with a length of 0.5 to 1 μm are already visible at the apical cell ends facing the inner surface of the plakea (Fig. 5q). Before the peripheral region of the plakea begins to curl, the basal ends of the cells at the posterior pole become pointed (Fig. 5b). Thus, the teardrop shape of these cells changes into a spindle (fusiform) shape (Fig. 10b).

#### Early inversion stage (Fig. 3b, c)

At the beginning of inversion the previously conical apical cell ends (Fig. 5a, b and q) in the bend region become hemispherical and, simultaneously, the basal cell ends taper (Fig. 3b, c). The cells in the peripheral region of the plakea rotate outwards and start moving “downwards” (in the direction of the posterior pole) (Fig. 3b, c). In this stage the cells at the posterior pole remain spindle-shaped and have a length of 8 to 10 μm and maximum width of 3 to 5 μm (Figs. 3b and 5c, r).

#### Mid-inversion stage (Fig. 3d-f)

During the mid-inversion stage, the plakea changes its shape from convex to concave (with respect to the apical cell ends) (Figs. 3d-f and 5d-f). During this process, the peripheral cells bend along their apical-basal axis without losing the contact with their neighboring cells (Figs. 3d and 5d, asterisks). The cells in the bend region form a fan-like structure. The apical cell ends of these cells continue to round up, the basal cell ends become even thinner and the elongation along the apical-basal axis of these cells continues. Furthermore, cells closer to the posterior pole of the plakea gradually undergo the same shape-shifting (Figs. 3d-f and 5d-f). The thinning of the basal ends is most prominent in the cells of the bend region (Figs. 3d, e, 5d and 6c). The cells in the center of the plakea, which corresponds to the posterior pole, reshape from a spindle to an ellipsoidal shape, i.e., both conical cell ends become hemispherical (Fig. 5e, f). Both cell wedging and cell elongation progress and the plakea becomes more and more bowl-shaped. Moreover, a transparent region appears at the apical part of each cell (Fig. 3e, f) indicating that the chloroplast no longer spans the entire cell.

#### Late inversion stage (Fig. 3g-h)

During the late inversion stage, the bowl-shape of the plakea develops into an ellipsoid-shape while the basal ends of all cells of the plakea become even more tapered (Figs. 3g, h, 5g and 6f, g). In the last third of the inversion process the cells continue to elongate. The movement of the cell sheet pauses about 15 min after the beginning of inversion when it has adopted the shape of an ellipsoid with an opening (phialopore), this opening still accounts for a sector of one third of the ellipse in lateral view (Figs. 3h and 5h). At this stage, all cells of the bowl-shaped plakea possess rounded (hemispherical) apical and basal cell ends and all cells are elongated along their apical-basal axis (Figs. 3g, h, 4a and 5g, h, n, s). The cells now have a length of 13 to 15 μm along their apical-basal axis and a maximum width of 3.5 to 5.5 μm. In cross sections perpendicular to the apical-basal axis the cells display a pentagonal or hexagonal outline (Fig. 5o). Overall the cells exhibit the shape of an elongated teardrop (Fig. 10c). Note that the ratio of basal-to-apical diameter is less than one in these elongated teardrop-shaped cells, but greater than one in teardrop-shaped cells before inversion. For the following 20–30 min in development, no further cell sheet movements and cell shape changes can be observed. It should be noted that the flagella of all cells grow slightly during inversion and exhibit a length of 2.5 to 3 μm at the end of inversion (Fig. 4a).

#### Post-inversion stage (Fig. 4a-d)

About 20–30 min after inversion has been completed, all cells of the embryo shorten simultaneously along their apical-basal axis and the apical cell ends flatten. The gaps between the apical ends of the cells become smaller until they disappear and the cells are in contact along their entire lateral surfaces. Cross-sections of the cells along their apical-basal axis show trapezoid outlines (Fig. 5i, t). Cross-sections perpendicular to their apical-basal axis show pentagonal or hexagonal contours (Fig. 5p). Hence, the cells possess the shape of a truncated pyramid (Fig. 10d) with a length of 6 to 8 μm along their apical-basal axis, a basal diameter of 1.5 to 2.5 μm and an apical diameter of 5 to 7 μm. After completion of inversion, the flagella rapidly grow and they reach a length of 12 to 15 μm about 30 min after inversion (Fig. 4b).

During the following development all cells gradually become rounder and change their shape from truncated pyramids to spheres (Figs. 4d, 5j, k and 10e). Only then, the phialopore eventually closes and the juvenile alga becomes spherical (Fig. 4c). Subsequently, all cells start to secrete ECM and thus, the juvenile alga grows in size and the distance between its cells increases. Shortly after the release of the young spheroid from its mother spheroid, the cells of the offspring have a diameter of 6 to 8 μm (Fig. 4d).

### Region and stage specific localization of CBs and other cellular details

The localization of CBs was analyzed using transmission electron microscopy. Figs. 6, 7, 8, and 9 show transmission electron micrographs of successive developmental stages.

#### Pre-inversion and early inversion stage

Before inversion begins, the CBs connecting the teardrop-shaped cells are located at the equator or near the apical end of the cells (Fig. 6a). Spindle-shaped cells at the posterior pole of the plakea are connected by CBs at the cell equator (Fig. 6b). In addition, transmission electron microscopy images show an apical localization of the cell nuclei, which have a diameter of about 2 μm (Fig. 6a). Cross-sections of flagella can also be observed in the hollow of the concave, bowl-shaped plakea (Fig. 6a) indicating that the biosynthesis of flagella starts before inversion. Further observations are that the cells usually contain several vesicle-like structures with a diameter of 1 to 1.5 μm (Fig. 6a, b), which resemble contractile vacuoles [19], and that the chloroplasts normally have more than ten starch grains (Fig. 6a, b).

#### Mid-inversion stage

The peripheral region of the plakea bends outward, the posterior pole moves towards the opening of the previously bowl-shaped cell sheet, and seven to eight minutes after the beginning of inversion, the plakea is almost flat. In this stage the cells in the bend region are connected by CBs at their basal ends (Fig. 6c).

#### Late inversion stage

In the late inversion stage both cross-sections along the apical-basal axis of the elongated teardrop-shaped cells and transverse cross-sections through the plakea show that in this stage all cells are connected to their neighbors at the basal cell ends (Fig. 6d-g). Of particular note is that some of the CBs are pervaded by rough endoplasmatic reticulum (Fig. 6e, main and inset). Besides, the flagellar ends of the cells now face outwards (Fig. 6d, f) and the embryonic vesicle is visible as an electron-dense line surrounding the plakea (Fig. 6d, f).

#### Post-inversion stage

After the transition from the elongated teardrop shape to the shape of truncated pyramids, the cells continue to be connected by CBs near their basal ends (Fig. 7a, b, main and inset). Despite the absence of CBs from the apical halves of the cells, the cells are closely packed exhibiting distances of 20 to 80 nm (Fig. 7a) between the cells. Both the apical and basal cell surfaces are quite flat and hardly rounded. During the transition to the shape of truncated pyramids, the cells begin with ECM biosynthesis and secretion (Fig. 7a, b). Subsequently, all cells shorten along their apical-basal axis and the ECM begins to appear in the form of distinct zones. As the process continues, the cells are separated from each other by electron dense ECM layers. Similarly to *Volvox carteri,* the ECM layers form honeycomb-like chambers around the cells (Fig. 8a, d). Subzones of the ECM, i.e., the boundary zone and the cellular zones 2 and 3 (Figs. 8a, d), were identified and named according to the nomenclature used in *Volvox* and its relatives [85, 86]. Figs. 8d and a show transverse and medial cross-sections of juvenile algae. The stage of the alga in Fig. 8d is slightly later in development than that of the alga in Fig. 8a. Due to the continuous increase in ECM after inversion, the distance between the apical cell surfaces and the outer edge of the boundary zone is larger by a factor of 1.5 to 2 in Fig. 8d compared to that in Fig. 8a. In the immediate area outside of the boundary zone, numerous cross-sections of flagella can be found in the transmission electron micrographs (Fig. 8a, d); some cross-sections are found at considerable distance from the boundary zone giving an indication of the length of the flagella which can reach up 15 μm. Even in this late post-inversion stage, the cells are still connected by CBs near their basal ends (Fig. 8a-e), but the CBs disappear during the following two hours. Once the juvenile algae are released from the embryonic vesicle and from the mother spheroid, their spherical cells no longer possess cell-cell connections. Each cell of the juvenile alga is already enclosed in a newly synthesized embryonic vesicle that is embedded in ECM (Fig. 9). At this stage the chloroplasts contain three to four times more starch grains than cells about 40 min after completion of the inversion process (Fig. 9).

### Modeling of cell shapes, CB locations and illustration of the entire inversion process

To model the different identified cell shapes, the outlines and dimensions of numerous cells before, during and after inversion were determined using light microscopy images of living samples, light microscopy images of semi-thin sections and transmission electron microscopy images of thin sections (see Methods). Based on these extensive data, we generated three-dimensional models of consensus cell shapes. We distinguish among teardrop-, spindle-, truncated pyramid- and elongated teardrop-shaped cells as well as spherical cells, the latter observed shortly after the release from the mother spheroid. Fig. 10 shows a surface-rendered three-dimensional representation of the generated cell shape models. These models illustrate that throughout inversion there are not only considerable changes in cell shape but also in the cell volume. Moreover, Fig. 10 shows for each cell shape the determined localization of the CBs. Special interest was paid to the position of CBs in relation to the apical-basal axis. Furthermore, we determined consensus curvatures of the entire cell monolayer throughout inversion using traced contours of plakeae from image series of in vivo studies such as that of Fig. 3. Finally, we developed a summary model for the complete process of embryonic inversion, which is shown in Fig. 11. The summary model illustrates the shape-shifting and dynamics of the cell sheet throughout inversion and thereafter. It also shows the localization of the observed cell shapes, the directions of cell layer movements, the relative position of the CB network and the length of flagella. The summary model is discussed below.

## Discussion

Among the inversion processes of extant volvocine species, the type-A and type-B inversions of spherical *Volvox* embryos involve the topologically most complex cell sheet deformations. Therefore, particularly these types of inversion have inspired scientists in the field to speculate about how such a major morphological event might have evolved from simpler processes [6, 36, 38]. With respect to the complexity of cell sheet deformations, the inversion process of *P. californica* ranges somewhere between those of *Gonium* and *Volvox*. This allows for comparisons and provides the opportunity to address the question, which differences on a cellular scale facilitate topologically more complex cell sheet deformations. In our study of *P. californica*, we identify basic mechanisms that seem to be common to all inversion processes of volvocine algae. We then seek to explain how different physical constraints of the cell sheets of volvocine embryos (due to different cell numbers and different shapes of the pre-inversion embryos) are overcome. We do so in revealing a gradual increase in complexity of the application of the same basic cellular mechanisms. The identified shape-shifting regimes of cell sheets may help in understanding other developmental processes involving cell sheet deformations.

### Hypotheses about intra- and inter-cellular forces during inversion of *P. californica*

#### Pre-inversion stage

Our observations of the initiation of inversion of *P. californica* embryos differ from the descriptions of Kazuko Kikuchi. As mentioned above, pre-inversion movements of the cell sheet have never been observed in our studies. In contrast to Kikuchi’s approach, our time-lapse recording guaranteed that one and the same embryo was observed throughout development in chronological order. The images published by Kikuchi do not all appear to show one and the same embryo [20], which raises the question whether Kikuchi’s image sequence actually shows successive developmental stages. It should be noted that movements of the cell layer preceding inversion have been observed and reconfirmed only in different species of the genus *Volvox* [39, 46, 53, 87]. These sporadic movements of the spherical cell monolayer, known as ‘denting’, occur as indentations that quickly (within minutes) appear and disappear between the cell division phase of *Volvox* embryos and the beginning of inversion. Aside from the unobserved pre-inversion movements of *P. californica* embryos [20], our observations regarding the overall deformation of the cell sheet are consistent with Kikuchi’s description.

As shown above, all cells of the *P. californica* plakea adopt a teardrop-shape before inversion begins (Fig. 11a). This cell shape resembles the ones described for pre-inversion embryos of numerous other volvocine species, namely *Eudorina elegans* [16], *V. carteri* [39], *V. tertius* [45], *V. globator* [53] and *V. rousseletii* [53]. In *V. rousseletii*, teardrop shapes were also observed in pre-inversion sperm packets [44]. In *V. carteri* and *V. globator*, filamentous actin was shown to be most abundant in the conical half of the teardrop-shaped cells indicating actin-dependent apical contraction of the cell [49, 53]. The shape resemblance of pre-inversion cells in numerous multicellular volvocine species could indicate an evolutionary conservation of the underlying shaping process among the multicellular volvocine genera.

Shortly before inversion of *P. californica* embryos starts and the cell sheet begins to curl, the basal ends of the cells at the centrally located posterior pole become pointed, while the other cells remain teardrop-shaped (Fig. 11b). Thus, not all cells undergo the same sequence of cell shape changes. Neither the basal nor the apical cell poles of the resulting spindle-shaped cells are in contact with any neighboring cells that might otherwise exert forces on them, indicating that their spindle shape is the result of active intracellular changes of the cytoskeleton. The biomechanical role of the spindle shape for the successful execution of inversion remains unclear. In the cases in which a transition to spindle shaped cells has been observed during inversion of species of the genus *Volvox*, this change in shape was accompanied by a thinning of the cells along their apical-basal axis. If interlinked cells of a cell sheet become thinner and if the connections are not elastic, the surface area of the cell sheet will contract as a consequence. However, in *P. californica*, we did neither observe that the spindle-shaped cells become thinner nor that the central region of the cell sheet with the spindle-shaped cells contracts. This suggests that, while contraction of the posterior region of the cell sheet is crucial to invert spherical *Volvox* embryos [49, 53, 54], it is not necessary to contract the posterior region of the bowl-shaped embryos of *P. californica*.

It is also worth mentioning that embryonic cells of *P. californica* already possess short flagellar stubs before inversion begins. In contrast to our observations, Kikuchi noted that the flagella do not begin to grow in *P. californica* until inversion begins [20]. Nevertheless, it is very unlikely that any beating movements of flagella contribute to the forces that drive inversion since inversion is not impeded in flagella-less mutants of *V. carteri* [88].

#### Beginning of inversion - outward curling of the edge

The inversion process begins when the peripheral region of the plakea curls outwards (Fig. 11). This cell sheet bending can be explained by the apical expansion and basal thinning of the peripheral cells along with the relocation of their CBs towards the basal cell poles (Fig. 11b, c). This combination of cell wedging and movement of CBs resembles the mechanism that drives cell sheet curling during inversion in the genus *Volvox* [51, 53]. However, instead of developing thin stalks, the cells that form the bend region in *P. californica* embryos merely reverse their apical-to-basal ratio of the cell diameter. It seems that the thinner and longer the basal cell ends are and the more the CBs move towards these ends, the smaller the possible minimum radius of curvature is.

When cell wedging is observed in an invagination region that is confined by surrounding cells (as is the case in type B inversion of *Volvox*), it cannot be excluded that the wedge shape of some cells might partially occur more or less passively due to the exerted forces of surrounding cells that bend the entire cell sheet. In contrast, due to the lack of adjacent cells, it seems geometrically impossible for cell wedging to occur at the edge of a cell sheet by means of extracellular forces; shape-shifting of peripheral cells must be caused by cell-intrinsic forces.

Interestingly, the outermost cells of the outward curling plakea stayed in close contact with their neighboring cells and did not fan out, despite the fact that CBs could only be observed at the basal cell ends. The close contact between cells on the one hand and the missing CBs on the lateral surfaces on the other hand, suggests the existence of further intercellular connections between neighboring cells of the inverting embryo. A reasonable candidate for such a function is the cell adhesion molecule Algal-Cam, which has been identified and characterized in *V. carteri* [89].

The force for relocation of the CBs during inversion of volvocine algae is likely provided by the motor protein InvA, which has been identified and characterized in *V. carteri* [51]. Homologs of the InvA motor protein have already been found in other volvocine species including *Pleodorina* [36], *Eudorina* [36], *Pandorina* [36], *Gonium* [36, 90] (GPECTOR_36g129, Acc. No. KXZ47277) and *Chlamydomonas* [51, 91]. Even the more distantly related InvA homolog of *Chlamydomonas*, IAR1, showed its functional efficiency: an inversionless *invA* mutant of *V. carteri* has been rescued by transgenic complementation using the *IAR1* gene of *Chlamydomonas* [51].

#### Implementation of inversion - a wave of cell wedging

The cell wedging and relocation of the CBs proceeds from the periphery to the center of the plakea (Fig. 11). This circular wave appears to create the force that causes the center of the plakea to move towards the opening of the bowl-shaped cell sheet and the periphery to move “downwards” (Fig. 11b-h). Most probably, the simultaneous cell elongation is mediated by polymerization of microtubules, just as in *V. carteri* [4, 39]. At the end of inversion, all cells are elongated teardrop-shaped and about 1.5-fold larger in volume than the cells at the beginning of inversion (Fig. 10c). This increase in volume coincides with an apical expansion of the cells, which is easy to observe: before inversion, the bowl-shaped chloroplasts seem to fill each cell and all cells are completely green; however, later when the apical region of the cells expands and the elongated teardrop-shaped cells emerge, a clear region appears in the cells that obviously does not contain any part of the chloroplast (Fig. 4a). The apical expansion might be mediated by formation or filling of apical vesicles, which are visible in thin sections (Fig. 6f). Similar vesicles have been observed in asexual reproductive cells of *V. carteri* [4]. Elongated teardrop-shaped cells also occur in inverting embryos of *Platydorina caudata* [23]; however, it should be noted that in the specified publication the cells are misleadingly referred to as spindle-shaped, even though the corresponding cells clearly have rounded apical cell halves.

#### Post-inversion stage

When all cells have adopted the elongated teardrop shape, the plakea resembles an ellipsoid with an opening (Fig. 11h). Both the shape of cell monolayer and the shape of the cells do not change for at least 15 min. Considering that the embryo retains an opening with a diameter of 30-40 % of the total diameter of the embryo and cell sheet deformations do not progress any further at that time, the inversion of *Pleodorina* embryos should not be referred to as “complete” at this stage in development.

After this period without visible changes, the cells adopt the shape of truncated pyramids (Fig. 11i, j), which resembles cell shapes in post-inversion embryos of *Pandorina* and *Eudorina* [38]. For both genera it was suggested that the characteristic cell shape is caused by pressure that the cells exert on each other [38]. However, such compressive forces would not sufficiently explain the flat apical surfaces of the cells. Since CBs can only be found at the basal cell ends, this shape is another indication for additional cell-cell-connections that bring also the apical cell ends close together and that are responsible for the flat shape of the apical cell ends.

Our transmission electron microscopy studies showed that once the cells adopt the shape of truncated pyramids, they continuously secret ECM, thereby increasing the lateral distance between the cells (Fig. 11j-l). Each cell is embedded in its own ECM compartment with honeycomb-like, electron dense walls. In some cases, we observed ECM compartments with proximal ‘floors’. Such closed ECM compartments with ‘floors’ are also known from all species of the sections Euvolvox and Merrilosphaera within the polyphyletic genus *Volvox* [4].

In *Pleodorina* the phialopore of an inverted juvenile alga does not close before all cells round up and the entire alga becomes spherical (Fig. 11k). Thus, there is a great delay before inversion is really completed in *Pleodorina*. Due to the continuous ECM secretion, the distance between the cell bodies continues to increase (Fig. 11). The newly synthesized ECM meshwork takes over the task of holding the cells in place making the CBs unnecessary at this stage in development and actually the CBs progressively disappear. It is unclear whether the CBs are actively retracted or whether they break passively due to the increasing distance between the cells.

During the transition from the elongated teardrop shape to the shape of a truncated pyramids and then to spherical cells, the difference between the apical-basal length and the diameter of the cells continuously decreases. These changes could be the result of a combination of an emergent anisotropy of the cytoskeleton, e.g., due to depolymerization of microtubules, and ongoing cell growth (Fig. 10d, e).

### Impacts of biomechanical constraints on the different inversion processes of multicellular volvocine algae

To investigate the origins of inversion and to identify basic principles of cell sheet deformations it is useful to include the least complex species among the volvocine algae. The minimum requirements for an organism to be able to perform cell sheet deformations are I.) to consist of more than a single cell and II.) to possess cell-cell connections that allow for force transmissions between the cells.

The simplest extant, multicellular volvocine species with CBs connecting the cells is *Tetrabaena socialis* (Fig. 1). *T. socialis* consists of only four cells, all of which possess reproductive capabilities. The observable CBs between embryonic cells of *T. socialis* are the result of incomplete cytokinesis [92]. However, so far inversion-like movements or cell shape changes have not been detected in *T. socialis*. Additional electron microscopic studies would be required to determine whether changes in the location of the cytoplasmic bridges occur in this species. With one exception, all volvocine genera with more than four cells perform some kind of cell sheet folding. The exception is the inversionless genus *Astrephomene* (Fig. 1), which quite fittingly means “not turning itself”. Amazingly, embryonic cells of *Astrephomene* divide in such a way that the flagellar, apical ends of all cells face the exterior right after cleavage.

In *Platydorina caudata*, the fully cleaved, pre-inversion embryos comprise a slightly concave plate of 16 cells with conical apical cell poles [23]. The cells are connected with each other by CBs that are localized close to their cell equators. The cells have been described to undergo uniform simultaneous shape changes during inversion. In this process, all basal cell ends become conical and the embryo itself adopts a hemispherical shape (see Figures 14–17 in [23]). At the end of inversion the CBs are located at the basal cell ends.

Pre-inversion embryos of *Gonium* consist of 16 cells that build a concave (with respect to the apical cell ends) cell sheet, or, in other words, a bowl with the apical ends (flagellar ends) at the inner surface. During inversion of *Gonium*, the concave curvature of the cell sheet decreases until it resembles an almost flat plate. Then, the sheet inverts its initial curvature and becomes slightly convex [18, 25, 38]. Before, during and after inversion the cells remain connected by CBs [18, 26, 27].

In the 16- to 32-celled *Pandorina* and the 32- to 64-celled *Eudorina*, pre-inversion embryos are likewise bowl-shaped and the first half of their inversion processes does resemble inversion in *Gonium* [38]. However, in *Pandorina* and *Eudorina* the inverting cell sheets do not only become convex but the cell movements continue until the embryos form spheres [16, 25, 37, 38]. Therefore, Kirk referred to this type of inversion process as ‘complete’ inversion. We observed in *Pleodorina* that there is a great delay before the phialopore of an inverted juvenile is closed and inversion is really completed. To our knowledge, such a delay before the phialopore is closed has not yet been reported for other inversion processes of volvocine algae.

According to the previous descriptions of *Gonium*, *Pandorina* and *Eudorina*, all cells of their embryos likewise undergo simultaneous and uniform cell wedging and the basal cell ends become conical [16, 18, 37, 38]. It appears that uniform and simultaneous cell shape changes are sufficient to fold the formerly flat cell sheet in *P. caudata* and the initially bowl-shaped embryos of *Gonium*, *Pandorina* and *Eudorina*.

In contrast, we observed non-simultaneous, non-uniform cell shape changes in the 64- to 128-celled *Pleodorina* embryos. Our observation suggests that *Pleodorina* embryos have passed a threshold in cell number and embryo size that requires a more complex inversion process. We observed in *Pleodorina* that the cell sheet curling was initiated at the peripheral region and then the bend region moved towards the center (the posterior pole) of the plakea. The cells showed successive shape changes beginning from the edge and moving towards the center of the plakea. Such a gradual curling of a bowl-shaped cell sheet from the edge to the center seems to be energetically more favorable compared to the application of simultaneous and homogeneous forces across the entire cell sheet. In the latter case, the surface area of the bowl would need to increase around the edges and/or to decrease at the central region. This might be achieved by active expansion or passive stretching at the periphery and/or active contraction or passive compression at the center (None of which we have observed in inverting *P. californica* embryos). It appears that a cell sheet with 16 to 32 cells is less confined and easier to invert than a cell sheet with 64 to 128 cells. This could explain why simultaneous shape changes are sufficient to drive inversion of embryos with a lower number of cells (less than ~60 cells) whereas embryos with larger cell numbers (more than ~60 cells) require temporally and spatially shifted cell shape changes.

Moreover, we observed that cells in different regions of the *Pleodorina* plakea even perform different sequences of cell shape changes. Only the cells at the center of the plakea passed through the stage with the spindle shape. This finding suggests that inversion in volvocine algae with larger cell numbers requires not only temporally shifted changes of cell shape but also region specific sequences of changes in cell shape. This then raises the interesting but difficult question about the cause for the differences in changes of cell shapes. The sequence of changes could depend on the position of the respective cell within the cell sheet and on the forces acting there. The regulation of local cell shape changes during tissue deformation has been the subject of numerous studies [80], but is so far poorly understood. Simple model organisms like *Pleodorina* might help to unravel the complicated interactions of cell differentiation, mechano-sensing and other mechanisms involved in tissue dynamics.

### Evolution of inversion in volvocine algae

The evolution of multicellularity in volvocine algae most probably involved incomplete cytokinesis from the very beginning [4, 36]. It is possible that incomplete cytokinesis resulted in a small cell sheet, e.g., with 4 to 8 cells, with a slightly concave curvature (with respect to the flagellar cell poles). The next step in evolution would have been that the slightly concave curvature developed into a slightly convex curvature during ontogenesis of the organism in order to improve its swimming performance. The improvement is due to the reduced interference between the flagella of different cells in a convex cell sheet as compared to a concave cell sheet. This assumed step in evolution resembles the development of *Gonium* embryos, which undergo quite a simple inversion process to expose the apical cell poles with the flagella (Fig. 2a). Later, volvocine algae with more and more cells evolved. Due to the primary importance of exposed flagella for motility, increasing cell numbers in the descendants of *Gonium*-like ancestors probably led to increasingly complex inversion processes. However, the phylogeny of volvocine algae is not as straightforward as one might be inclined to think.

Originally, the classification of the volvocine genera within the order Volvocales was simply based on morphological traits observed by standard light microscopy [93–95]. From the 1980s onwards, however, different molecular phylogenetic studies showed that most genera within the Volvocales are polyphyletic and that the different body plans evolved several times independently [4, 58, 96–101]. A recent phylogenetic tree based on the nucleotide sequences of five chloroplast genes is shown in Fig. 1 [6, 35, 55–57]. Molecular phylogenetic analyses revealed that the type of body plan found in the polyphyletic genus *Pleodorina* has evolved independently at least twice after the divergence of lineages with body plans such as in the extant *Gonium*, *Pandorina* and *Eudorina* [4–8, 35, 55]. In terms of organismal complexity, *P. californica* is classified between the genera *Eudorina* and *Volvox* (Fig. 1, Additional file 1). Each lineage that led to a member of the genus *Volvox*, which is also polyphyletic, nevertheless descends from a common unicellular ancestor (Fig. 1). It has been hypothesized that in each lineage intermediate forms of increasing complexity (cell-number, increasing ECM, germ-soma differentiation) might have existed [36].

Most probably, members of the genus *Volvox* evolved from a *Pleodorina*-like ancestor with bowl-shaped embryos. Despite the fact that *Volvox* embryos consist of several thousand cells forming a spherical monolayer before and after inversion, the inversion process of the bowl-shaped *P. californica* plakea does involve characteristics of the two inversion types A and B known from different *Volvox* species. During type A inversion of *Volvox* embryos (e.g., in *V. carteri*) the four ‘lips’ at the phialopore at the anterior pole of the spherical cell sheet curl backwards and the cell sheet glides over the posterior hemisphere. By contrast, type B inversion (e.g., in *V. globator)* is characterized by the appearance of a circular invagination at the equator of the embryo, an ‘upwards’ movement of the posterior hemisphere into the anterior hemisphere and a widening of the circular phialopore to allow for gliding of the anterior hemisphere over the posterior hemisphere.

In *Pleodorina*, both the outward curling of a free edge at the beginning of inversion and the wave of cell shape changes that propagates towards the posterior pole resembles the type A inversion process. However, before inversion begins, all cells of a *P. californica* plakea are teardrop-shaped and only part of the cells (localized at the posterior pole) become spindle-shaped, just as in the type B inversion process. At the end of both types of inversion processes in the genus *Volvox*, all cells of the embryos adopt a columnar shape resembling an elongated prism with a hexagonal or pentagonal cross-section [39, 53]. By contrast, *P. californica* cells never adopt such a shape. In *P. californica* embryos, the shape that comes closest to the columnar cell shape of *Volvox* embryos is the shape of a truncated pyramid. Possibly, the non-parallel cell walls of truncated pyramid-shaped cells are resulting from the diameter of the spherical post-inversion embryo, which is significantly smaller in *P. californica* than in *Volvox*. In *P. californica*, the closure of the phialopore and transformation of the juvenile alga into a sphere takes place a significant time after the process of inversion. It is only then that the spherical cells emerge, which no longer possess CBs and which gradually move away from their neighbors due to ECM secretion and expansion of the spheroid.

In evolution, A and B types of inversion developed several times independently from each other (Fig. 1). It is unclear whether one of the two inversion types evolved first. However, the question arises which changes to the inversion process of *Pleodorina* could result in type A and which changes could result in type B inversion in *Volvox*. Following from the above, type A inversion may be seen as the elaboration of the outward curling of the free edge of the embryo while type B could be an elaboration of the spindle shaped cell shape changes in the posterior of the embryo.

Unfortunately, there are no fossils of ancestral or intermediate forms within the Volvocales, which might otherwise help to resolve lines of ancestry. However, the Volvocales are rich in closely related extant species that conveniently serve as models to learn how body plans, biophysical constraints and morphological processes led to convergent evolution of similar features in different lineages.

## Conclusions

In conjunction with finding from previous reports, this study shows that the most basic and general mechanisms underlying all forms of cell sheet deformation in multicellular volvocine algae are (i) active cell shape changes and (ii) active movements of cells relative to the CBs connecting them (Fig. 11).

Compared to species with lower or higher cell number, inversion in *P. californica* shows intermediate complexity. With increasing cell number of multicellular volvocine genera there is a trend towards more complex inversion processes. Not only does the inversion of the cell sheet become more complex but also the appearing cell shapes show increasingly more spatially and temporally distinct differences and different cell shapes occur in different regions of the cell sheet.

There are differences between the inversion processes of bowl-shaped and spherical embryos not only regarding the sequences of the cell sheet deformations. Compared to a spherical embryo, inversion of a bowl-shaped embryo involves less dramatic cell shape changes, e.g., no stalk-formation and no flattening of cells (as in *Volvox*, type B).

The number of cells in a bowl-shaped embryo has consequences for the temporal progression of cell shape changes during inversion. In a bowl-shaped embryo with a lower number of cells, as found in *Pandorina* and *Eudorina*, simultaneous shape changes are sufficient to drive inversion, whereas in a bowl-shaped embryo with a larger number of cells, as in *Pleodorina*, temporally and spatially shifted cell shape changes are required.

Future research in inversion of multicellular volvocine algae should focus on the question of whether cell differentiation, mechanical signals or any other factors effect that sequences of cell shape changes show local differences within the same cell sheet.

Seen in a broader perspective, comparative studies of inversion in multicellular volvocine algae might help to understand the basic principles and underlying mechanisms that drive cell sheet deformations in multicellular organisms. Due to their relative simplicity, multicellular volvocine embryos are particularly suited to inspire and experimentally verify mathematical models for developmental processes. The first mathematical model of type B inversion in *Volvox* has recently been published [54]. Comparative modelling of inversion processes in related species might give further insight into the mechanical constraints that differently shaped cell sheets are subject to.

## Methods

### Strain and culture conditions

The wild-type *Pleodorina californica* strain SAG 32.94 was obtained from the Sammlung von Algenkulturen der Universität Göttingen (Culture Collection of Algae at the University of Göttingen, SAG), Germany [102]. Strain SAG 32.94 was collected from a drainage ditch near to Glen Burnie, MD 21060, United States in 1993 by Franklyn D. Ott. Cultures were grown in Jaworski’s medium [103–105] at 28 °C under a cycle of 8 h dark/16 h light [106] with an average of approximately 100 μmol photons m^−2^ s^−1^ of photosynthetically active radiation. Small cultures were grown in a growth chamber in 10 mL Jaworski’s medium in glass tubes with caps that allow for gas exchange. Larger cultures were grown in 400 mL Jaworski’s medium in Erlenmeyer flasks aerated with sterile air at 150 cm^3^ min^−1^.

### High-resolution in vivo stereo light microscopy

Embryos with intact embryonic vesicles were mechanically removed from their mother spheroid up to 3 h before the onset of inversion. For this purpose, 5 ml of a *P. californica* culture were passed through a syringe needle with a diameter of 0.4 mm. High-resolution in vivo stereo light microscopy was performed using a motorized and automated Leica MZ16A stereomicroscope (Leica Microsystems, Wetzlar, Germany) with fully apochromatic optics and a transmitted light illuminator with cold light sources [38]. In this system, a resolution of up to 840 Lp mm-1 (=0.6 μm) was obtained. For higher magnifications, an Axioskop 40 FL upright microscope (Carl Zeiss, Oberkochen, Germany) equipped with Achroplan, Fluar and Plan-Neofluar objectives of up to 100× magnification was used. A digital PowerShot S50 camera (Canon, Tokyo, Japan) with a1/1.8” charge-coupled device sensor was used for photographic documentation.

### Fixation and dehydration of embryos

For light microscopy of sections and for transmission electron microscopy, spheroids were fixed as described previously [39] with the following modifications. Fixation was performed in 5 % glutaraldehyde (Agar Scientific, Stansted, UK) in Jaworski’s medium [103–105] for approximately 16 h. Specimens were washed three times with 50 mM phosphate buffer (pH 7.0) for 10 min each and post-fixed in 2 % osmium tetroxide (Electron Microscopy Sciences, Hatfield, PA) for 1 h. For light microscopy of sections and for transmission electron microscopy, the fixed spheroids were dehydrated by passage (30 min each) through 30 %, 60 %, 90 % and 100 % ethanol and then 100 % ethanol again. For light microscopyof sections and for transmission electron microscopy, the dehydrated specimens were infiltrated in a 1:1 solution of TAAB transmit resin (Agar Scientific) to ethanol (v/v) for 1 h, followed by a 2:1 solution of TAAB transmit resin to ethanol (v/v) for approximately 16 h. After evaporation of the ethanol, specimens were infiltrated twice in pure TAAB transmit resin in an evacuated desiccator for 2 h each. Specimens in resin were transferred to BEEM capsules (Agar Scientific), and the resin was then polymerized through a 16-h exposure at 70 °C. The polymerized blocks were removed from the BEEM capsules, and most of the excess resin was trimmed away with a razorblade under a stereo-microscope.

For light microscopy, sections with a thickness of approximately 2 μm were cut with glass knives on a Reichert-Jung Ultracut E ultramicrotome (Reichert Microscope Services, Depew, NY, USA) and transferred to glass slides. For transmission electron microscopy, sections with a thickness of approximately 80 nm were cut with a diamond knife (6620 SU; MicroStar Diamond Knives, Huntsville, TX, USA) on the Ultracut E ultramicrotome and mounted on 400 mesh copper grids (Plano).

### Staining

Sections for light microscopy (approximately 2 μm in thickness) on glass slides were stained for light microscopy by incubating the slides in 0.1 % toluidine blue (Sigma-Aldrich) for 10 s on a Combimag RCT hot plate stirrer (IKA-Werke, Staufen, Germany) at 70 °C. Thereafter, the slides were rinsed in deionized water; the sections were covered with droplets of deionized water; and the slides were placed on the hot plate stirrer until the water had evaporated. The sections were then mounted in Entellan rapid mounting medium (Merck, Darmstadt, Germany). Sections for transmission electron microscopy (approximately 80 nm in thickness) on grids were stained with 0.1 % uranyl acetate (Plano) for 10 s, washed by dipping them in deionized water, stained with 2 % lead citrate (Agar Scientific) for 10 s, and washed again with deionized water.

### Light microscopy and transmission electron microscopy of sections

Toluidine blue-stained sections (approximately 2 μm in thickness) of embryos were analyzed with an Axioskop 40 FL upright microscope (Carl Zeiss) using oil immersion objectives. The total magnification for light microscopy was up to 1000×. Heavy metal-stained sections (approximately 80 nm) were examined with a Hitachi H-500 transmission electron microscope (Hitachi, Tokyo, Japan) operated at 75 kV and with a Philips CM-100 transmission electron microscopy (Philips, Eindhoven, Netherlands) operated at 80 kV. The total magnification for transmission electron microscopy ranged from 3600 × to 40,000 ×.

### Two- and three-dimensional modelling

Light microscopy and transmission electron microscopy images of intact and sectioned embryos before, during and after inversion were adjusted to the same magnification. In this adjustment, shrinkage during processing (for example, through fixation and dehydration) was taken into account and corrected with the help of images of live material. Then the images were screened for the characteristics of interest and all appropriate images with a non-ambiguous assignment to a certain area of the cell monolayer were included in the further analyses. For each appropriate image, outlines of cells and nuclei, the position of CBs relative to the cell and the curvature of the cell monolayer were plotted on individual layers using Photoshop CS5 software (Adobe Systems, San Jose, CA, USA). Moreover, dimensions of structures (e.g., diameters and lengths) and arithmetical means thereof were determined. Based on these extensive data, we generated consensus cell shapes. For this purpose, at least 30 contours of the same anatomical structures were laid on top of the others to develop two- and three-dimensional consensus shapes. Three-dimensional wireframe modeling and texturing was performed using Blender (version 2.49b) software (Stichting Blender, Amsterdam, Netherlands). Two-dimensional vector graphics were developed using CorelDRAW GraphicsSuite X5 (version 15) software (Corel, Ottawa, Canada). Finally, we distinguish between teardrop-, spindle-, truncated pyramid- and elongated teardrop-shaped cells as well as spherical cells, which occur shortly after the release from the mother spheroid. All contours of cells were assigned to these cell shape categories.

## Additional files

Additional file 1: The algae of the volvocine lineage. (PDF 3075 kb)

## Abbreviations

CB: Cytoplasmic bridge
ECM: Extracellular matrix
