# Supplementary material for: Distinct shape-shifting regimes of bowl-shaped cell sheets – embryonic inversion in the multicellular green alga Pleodorina
Source: BMC Dev Biol. 2016 Oct 13;16:35. doi: 10.1186/s12861-016-0134-9 (PMC5062935; doi:10.1186/s12861-016-0134-9)
Supplement: Additional file 1: — The algae of the volvocine lineage. (PDF 3075 kb) [file 12861_2016_134_MOESM1_ESM.pdf]

Additional file 1:

The algae of the volvocine lineage

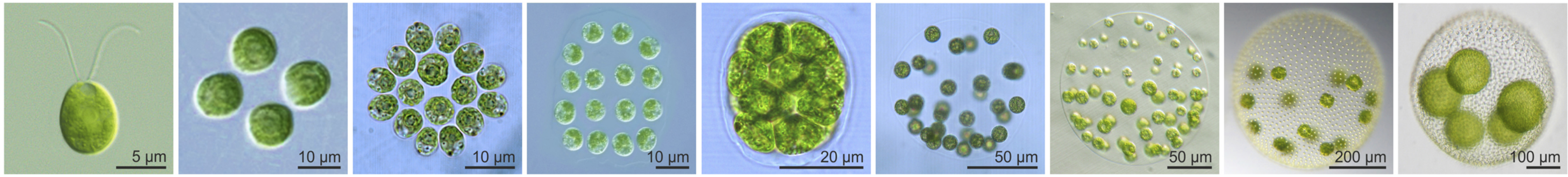

	Chlamydomonas reinhardtii	*Tetrabaena socialis*	*Gonium pectorale*	*Platydorina caudata*	*Pandorina morum*	*Eudorina unicocca*	*Pleodorina californica*	*Volvox carteri*	*Volvox globator*
Cell number	1	4	8-16	16-32	16-32	32-64	64-128	1000 - 8000	3000 - 40,000
Diameter [µm], adult	~10	~25	~30	~40	~30	~100	~150	500-2000	500-1500
Incomplete cytokinesis		✓	✓	✓	✓	✓	✓	✓	✓
Extracellular matrix	+	++	++	++	++	++++	++++	+++++	+++++
Germ-soma differentiation				+		±	+++	+++++	+++++
Embryonic inversion			+	++ [3]	+++	++++	+++++	+++++	+++++
Embryo shape before inversion			bowl, concave	bowl	bowl	bowl	bowl	spheroid	spheroid
Embryo shape after inversion			bowl, convex	double layer	spheroid	spheroid	ellipsoid [2]	spheroid	spheroid
Adult shape			bowl, convex	plate	spheroid	spheroid	spheroid	spheroid	spheroid
Non-uniform cell shape changes [1]							✓		✓
Free edge at bend region							✓	✓	
Wave of cell shape changes							+++	+++++	+++++
Flask-like cells				++				+++++	+++++
Re-location of cytoplasmic bridges			+	+++			+++	+++++	+++++

Increase in complexity

The relatives in the volvocine lineage range from unicellular forms to colonial and multicellular forms with increasing complexity. Nine representative species with characteristic developmental traits were arranged such that there is a progressive increase from left to right in morphologic and developmental complexity [1-4]. Check marks indicate that a given trait is present in the respective species. Graded differences in a given trait are indicated by 1-5 plus signs, and ± indicates ambiguity or occasional occurrence. The photomicrographs show *Chlamydomonas reinhardtii, Tetrabaena socialis, Gonium pectorale*, *Platydorina caudata*, *Pandorina morum*, *Eudorina unicocca*, *Pleodorina californica*, *Volvox carteri* and *Volvox globator*. The phylogenetic position of these nine species is shown in the evolutionary tree in Fig. 1 of the main text.

[1] Different groups of cells undergo different sequences of cell shape changes during inversion.

[2] *P. californica* embryos have the shape of an ellipsoid with a wide opening (phialopore) right after inversion; the phialopore does not close before all cells round up and the entire alga becomes spherical, i.e., there is a great delay before inversion is really completed in *P. californica*.

[3] Inversion is followed by intercalation in *Platydorina caudata* [5].

References:

1. Kirk DL. *Volvox*: molecular-genetic origins of multicellularity and cellular differentiation, Cambridge: Cambridge University Press; 1998.
2. Kirk DL. *Volvox* as a model system for studying the ontogeny and phylogeny of multicellularity and cellular differentiation. J Plant Growth Regul. 2000;19:265-274.
3. Prochnik SE, Umen J, Nedelcu AM, Hallmann A, Miller SM, Nishii I et al. Genomic analysis of organismal complexity in the multicellular green alga *Volvox carteri*. Science. 2010;329:223-226.
4. Hallmann A. Evolution of reproductive development in the volvocine algae. Sex Plant Reprod. 2011;24:97-112.
5. Iida H, Nishii I, Inouye I. Embryogenesis and cell positioning in *Platydorina caudata* (Volvocaceae, Chlorophyta). Phycologia. 2011;50:530-540.
